# Ultra‐early stage lower‐grade gliomas: How can we define and differentiate these easily misdiagnosed gliomas through intraoperative molecular diagnosis

**DOI:** 10.1111/cns.70044

**Published:** 2024-10-09

**Authors:** Zhe Han, Qingtong Wang, Jia Li, Huizhong Chi, Caizhi Ma, Deze Jia, Mei Qi, Xueen Li, Kailiang Zhang, Zichao Feng, Hao Xue, Gang Li

**Affiliations:** ^1^ Department of Neurosurgery, Qilu Hospital Cheeloo College of Medicine and Institute of Brain and Brain‐Inspired Science, Shandong University Jinan China; ^2^ Shandong Key Laboratory of Brain Health and Function Remodeling Jinan China; ^3^ Department of Pathology Shandong University Qilu Hospital Jinan Shandong China; ^4^ Department of Pathology Shandong University School of Basic Medical Sciences Jinan Shandong China

**Keywords:** gliosis, IDH mutation, intraoperative rapid molecular detection, ultra‐early stage lower‐grade glioma

## Abstract

**Background:**

Some lower‐grade gliomas (LGG) are difficult to distinguish morphologically from glial cell proliferation or inflammatory changes during surgery, leading to a high risk of incorrect diagnosis. It is crucial to differentiate between the two for making surgical decisions. We define these critical cases as “ultra early stage lower‐grade gliomas (UES‐LGG)”.

**Methods:**

We analyzed 11 out of 13 cases diagnosed with “gliosis” or “inflammatory changes” during surgery who tested positive for isocitrate dehydrogenase (IDH). Additionally, we conducted qRT‐PCR detection on 35 samples diagnosed with LGG during surgery and analyzed their DNA content within an effective circulating threshold range to infer the critical value between UES‐LGG and LGG. We conducted experiments using five standardized samples to infer the limited range of accurate detection of UES‐LGG during surgery.

**Results:**

In the comparative analysis of 11 samples and 35 samples, it was found that while there was no significant difference in the average DNA detection concentration between the two groups (159.36 ± 83.3 ng/μL and 146.83 ± 122.43 ng/μL), there was a notable statistical variance in the detection threshold for positive mutations (31.78 ± 1.14 and 26.14 ± 2.69, respectively). This suggests that the IDH mutation rate may serve as an indicator for differentiation between the two groups. Subsequently, DNA was extracted from standardized IDH mutant samples and subjected to gradient dilution for detection purposes. The results indicated a consistent increase in detection threshold as detection concentration decreased. When the detection concentration fell below <0.1 ng/μL, it became impossible to carry out effective threshold range detections. To further identify the precise detection interval, we conducted gradient division once again and sought to simulate the functional relationship between DNA copy number and cycle threshold within this interval. The research revealed that when the minimum detection concentration exceeded 250 copies/μL, a 100% detection rate could be achieved.

**Conclusions:**

This article defines UES‐LGG as a tumor type easily misdiagnosed in clinical practice due to its extremely low positivity rate during surgery. The popularization of qRT‐PCR based intraoperative molecular diagnosis greatly reduces errors caused by manual detection and improves disease detection rates during surgery. It provides a theoretical basis for more accurate surgical plans for surgeons.

## Graphical abstract

During surgery, we can perform rapid molecular diagnosis on samples with pathological diagnosis of gliosis or inflammatory changes, and identify Ultra‐early stage lower‐grade gliomas based on IDH mutations. This article attempts to discuss the criteria for defining Ultra‐early‐stage lower‐grade gliomas.

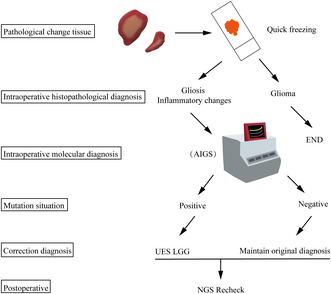

## 1 INTRODUCTION

Glioma is the most common intracranial malignant tumor, and some lower‐grade gliomas (LGG) have a high degree of differentiation, making it difficult to distinguish them from glial cell proliferation or inflammatory changes in morphology,^1^ especially for rapid examination during surgery. Pathologists need to distinguish the samples in a relatively short time. So some studies have shown that in the diagnosis of brain tumors, the perfect match rate between intraoperative rapid frozen sections and postoperative histopathological examination is only 53.8%, while the perfect mismatch rate is as high as 16.2%.^2^ Misdiagnosis during surgery may have a significant impact on the treatment and prognosis of patients. Recently, an increasing number of molecular markers have been proven to be crucial for the classification, typing, grading, prognosis, and treatment of gliomas. The fifth edition of the World Health Organization Classification of Central Nervous System Tumors, published in 2021, integrates the histological characteristics and molecular phenotypes of tumors and proposes new tumor classification standards.^3^ It is worth noting that molecular markers are indispensable in the diagnosis of almost all tumors. Among them, isocitrate dehydrogenase (IDH) mutations are crucial for the diagnosis and prognosis of gliomas, especially LGG, and are the key to distinguishing between LGG and glial cell proliferation.^3^

**FIGURE 1:**
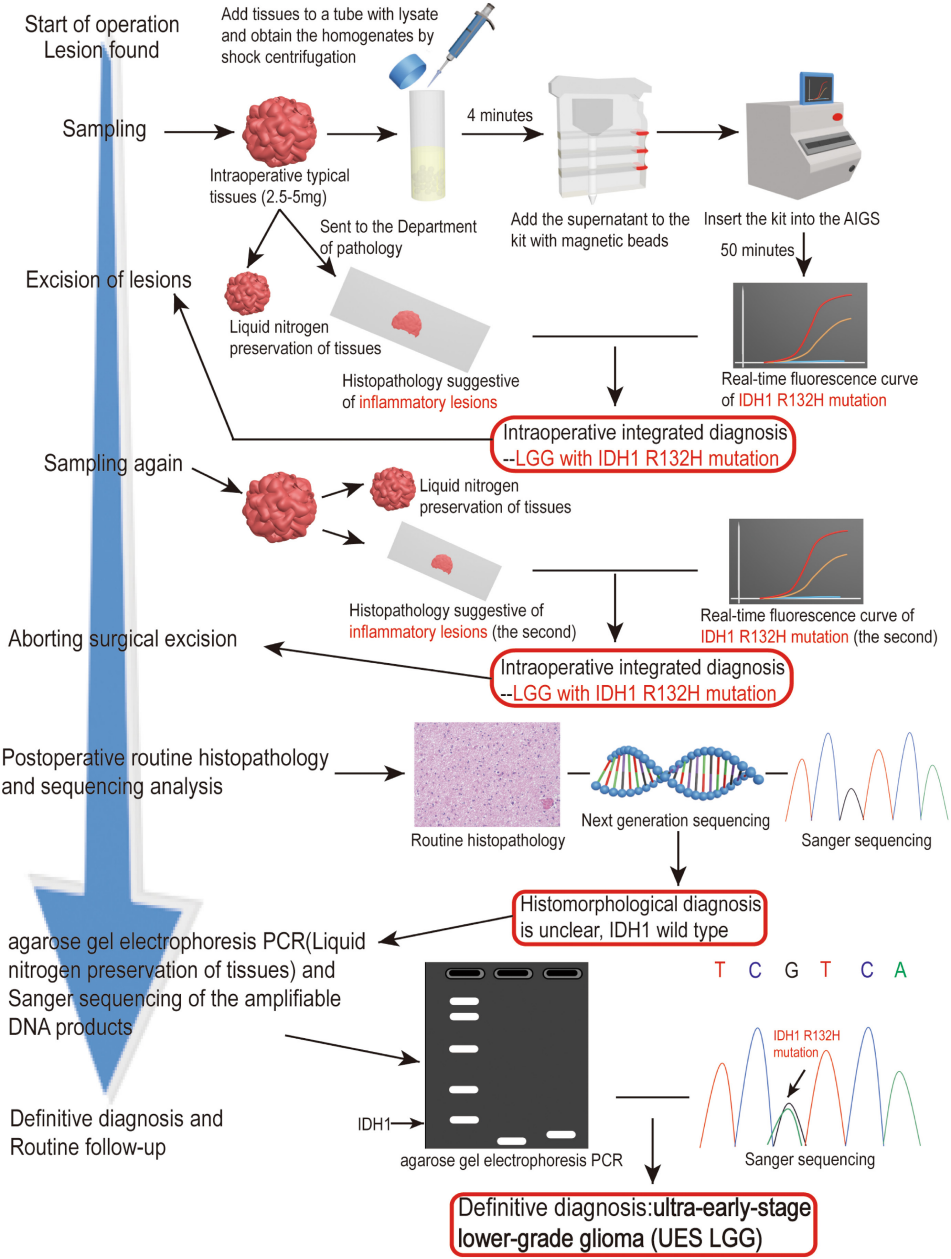
Based on practical cases, summarize the entire process of identifying and diagnosing UES‐LGG using intraoperative rapid molecular detection technology.

In clinical practice, we have found that a considerable number of LGGs are not resected according to the principle of tumor resection due to intraoperative diagnosis of “gliosis or inflammatory changes”, which leads to the progression of the tumor in the later stage or the need for secondary surgery, which only increases the patient's pain and wastes medical resources. However, these tumors were still unable to detect positive mutations of IDH with diagnostic significance through histopathological detection methods such as immunohistochemistry after surgery. In some cases, even after next‐generation sequencing, there were no results, and only more accurate real‐time fluorescence quantitative PCR was used to detect positive mutations. Therefore, for gliomas with extremely low tumor cell content and gene mutation abundance, we identify them as the early stage of LGG or referred to as ultra‐early stage lower‐grade gliomas (UES‐LGG). In this study, we first shared the process of discovering and treating UES‐LGG with the informed consent of a patient (Figure 1).

Obviously, UES‐LGG places high demands on the sensitivity and accuracy of molecular detection methods, and only accurate judgments during surgery can provide important guidance. In previous studies, we developed a fully automated nucleic acid detection and analysis system (AIGS) based on real‐time fluorescence PCR technology, which has been validated to have high detection sensitivity and accuracy.^4^ Therefore, in this study, we attempted to use this technology to identify UES‐LGG and describe it within a certain detection range. We hope that with the advent of the molecular diagnostic era, Advanced technology will enable us to make more accurate diagnoses and treatments.

## 2 CASE PRESENTATION

**FIGURE 2:**
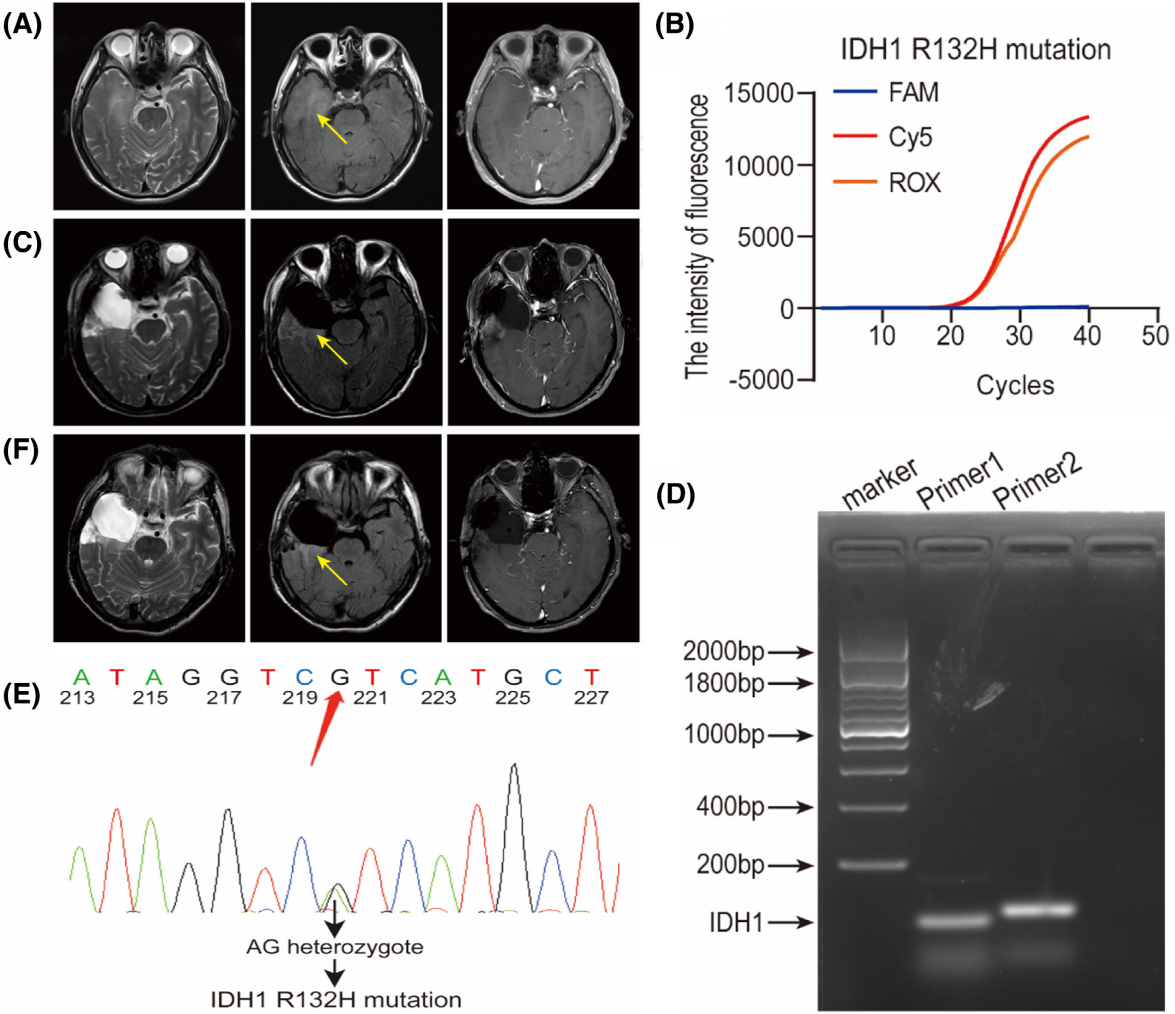
Imaging information and molecular mutation detection results of patients with UES‐LGG at different stages. The preoperative cranial MRI of the patient confirmed the location of the lesion (A). Perform an MRI examination 6 months after surgery (B). The MRI examination conducted 1 year after surgery did not show significant progression of the lesion (C). AIGS real‐time fluorescence PCR intraoperative detection result graph. ROX represents IDH1 R132H mutation, FAM represents IDH1 R132C/G/S mutation, and Cy5 represents GAPDH internal reference (D) in the real‐time fluorescence curve of IDH1. IDH1 agarose gel electrophoresis polymerase chain reaction (E) of pathological tissue. Primer1 represents Thermo Scientific™ O'RangeRuler™ 200 bp DNA ladder diagram (SM0633), and Primer2 represents the DNA ladder diagram used by the Automated Integrated Gene Detection System (AIGS). IDH1 Sanger sequencing (F) of diseased tissue.

On September 16, 2022, a 66‐year‐old male was admitted to the hospital due to paroxysmal limb twitching and dizziness. Brain MRI shows a space‐occupying lesion in the right frontal temporal insula (Figure 2A). Four days later, we underwent a right frontal temporal insular lesion resection surgery. During the surgery, we divided the tumor samples into two parts and performed intraoperative frozen tissue pathology testing and AIGS‐based IDH mutation detection. Approximately 30 min later, the intraoperative rapid frozen tissue pathological examination revealed mild microglial proliferation and inflammatory lesions were not ruled out. After another 15 min, AIGS detection showed the presence of IDH1 R132H mutation (Figure 2B). Although intraoperative frozen tissue pathology is not conducive to the diagnosis of glioma, considering the importance of IDH mutations in glioma diagnosis, we identified it as an LGG and decided to undergo a standard surgical resection. Postoperative routine histopathological examination showed proliferation of glial cells and IDH−, and surprisingly, NGS sequencing also indicated wild‐type IDH. Six months after surgery, the MRI results showed no significant progress (Figure 2C). In summary, we can find that no IDH mutation was found in the intraoperative histopathological examination and postoperative immunohistochemical testing of the patient, while our AIGS test reported a positive result. To further support our results, we carried out lipopolysaccharide gel electrophoresis PCR and Sanger sequencing verification on the samples in the laboratory after the operation, and the results showed that there was IDH1 R132H mutation (Figure 2D,E), The patient was ultimately diagnosed with astrocytoma, IDH mutation type. Based on this, we believe that this type of tumor is the early stage of LGGs, which can be referred to as “UES‐LGG”. At the last follow‐up (November 16, 2023), the patient showed no signs of progression (Figure 2F).

When there was no intraoperative molecular diagnosis before, we would terminate the surgery for cases with intraoperative histopathological diagnosis of “gliosis”, as most cases of gliosis do not require surgical treatment, which leads to some patients progressing to glioma years later.

## 3 MATERIALS AND METHODS

In response to the above clinical issues, we collected 13 case samples with intraoperative histopathological diagnosis of “gliosis” or “inflammatory changes”. At the same time, we also included 35 case samples with clear intraoperative and postoperative diagnoses of LGG during the same period. All patients underwent surgery at the Neurosurgery Department of Qilu Hospital, Shandong University from July 2021 to July 2023, and all patients underwent intraoperative tissue pathology testing, intraoperative AIGS rapid molecular pathology testing, postoperative immunohistochemistry pathology testing, and postoperative next‐generation sequencing testing, with the next‐generation sequencing results used as the gold standard for mutation detection. Informed consent was obtained from patients before sample collection.

All included samples were subjected to quantitative detection using an ultra‐micro UV visible spectrophotometer before loading, and amplification threshold detection was performed using a real‐time fluorescence quantitative PCR instrument. The results were compared and analyzed to observe whether the difference in DNA content between the LGG group (Group 1) and the “UES‐LGG” group (Group 2) was statistically significant, in order to speculate the possible DNA content boundary between the two groups. At the same time, in order to verify the accuracy of AIGS in detecting UES‐LGG during surgery, we selected five glioma samples that have been diagnosed with IDH mutations. After measuring the DNA sample content separately, the samples were subjected to gradient dilution, and after dilution, AIGS testing was performed again to determine the lower limit of detection within the effective threshold range. To determine the minimum concentration for AIGS detection rate of 100%, we further divided the DNA samples from<0.1 ng/μL to 10 ng/μL into gradients and determined the detection rate for each gradient.

In this study, we summarized a detection process based on the diagnosis and treatment process of case patients to identify and define tumors that are easily misdiagnosed (Figure 1). First, we sampled and tested the tumors discovered during surgery. When rapid histopathological examination showed “gliosis” or “inflammatory changes” during surgery, if the molecular diagnosis was an IDH mutation, we should diagnose it as “LGG”. The IDH mutation should be surgically removed according to glioma, and we should take samples from multiple locations for retesting to avoid histopathological diagnosis errors caused by tumor heterogeneity. After surgery, we routinely perform immunohistochemical pathological testing. If the result is IDH− and the final diagnosis is still “gliosis” or “inflammatory changes,” we can further perform PCR and sequencing verification. If the result is IDH mutation, we ultimately consider the type to be UES‐LGG.

All data analysis was conducted using SPSS 13.0 software (SPSS Inc., USA) and Graphpad Prism 10.1.2 (Graphpad Soft, USA). Continuous variables were presented as Mean ± SD. A normality test was performed on continuous data using the Kolmogorov–Smirnov/Shapiro Wilk test method. For samples that meet the criteria for a normal distribution, an independent sample *t*‐test was utilized to compare intergroup differences; otherwise, a Wilcoxon rank sum test was employed for comparison. Statistical significance was set at *p* < 0.05.

## 4 RESULTS

**FIGURE 3:**
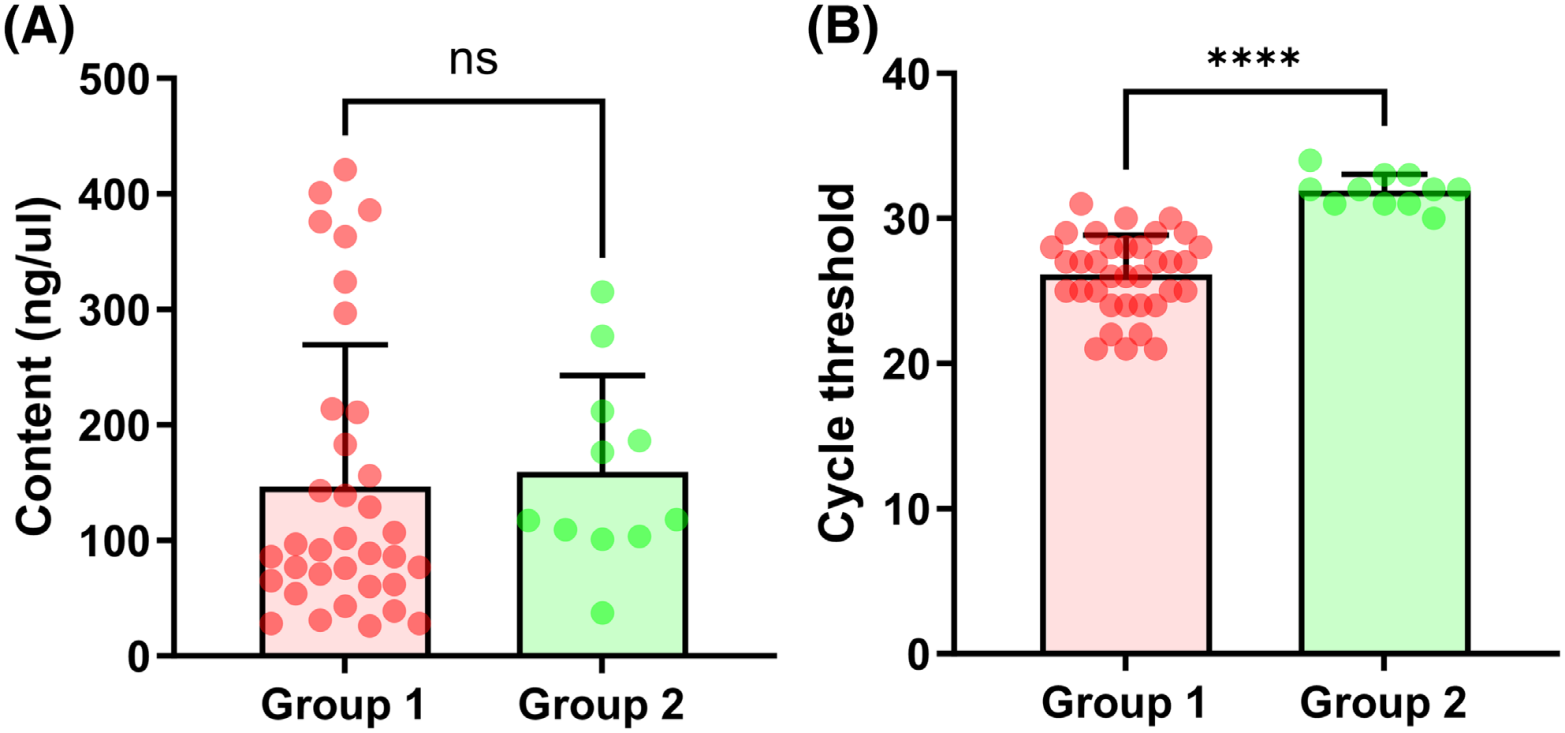
Comparison of AIGS detection results between the lower‐grade glioma group and the UES‐LGG group. Comparison of two sets of DNA concentration values (A). Comparison of two sets of circulating thresholds (CT) (B). Group 1: 35 cases of LGGs, Group 2: 11 cases of UES‐LGG.

The results of this study found that 11 out of 13 samples diagnosed with “gliosis” or “inflammatory changes” during surgery showed IDH mutations in AIGS testing. At the same time, we retained these 13 samples for postoperative next‐generation sequencing validation (Table 1) and obtained the same results, indicating that the accuracy of AIGS detection is as high as 100%. Based on their clinical information, 11 out of these 13 patients had their final diagnosis changed due to molecular diagnostic results. At the last follow‐up, only one patient experienced a recurrence, and all patients survived (Table S1). We took samples from these 11 mutated patients (with a volume size of “rice grains”) for DNA concentration testing, we found that the average detection concentration was 159.36 ± 83.3 ng/μL, while the average detection concentration of the 35 LGG groups was 146.83 ± 122.43 ng/μL. The difference between the two was not statistically significant (*p* > 0.05) (Figure 3A), but the circulating thresholds were 31.78 ± 1.14 and 26.14 ± 2.69, respectively. There was a statistical difference between the two before (*p* < 0.001) (Figure 3B), which means that these clinically difficult‐to‐distinguish types are not due to the low content of DNA samples being tested, which cannot be distinguished. The detection of effective mutations is due to the low abundance of gene mutations caused by the small number of tumor cells, so more cycles are often required for PCR amplification to be effectively detected.

**TABLE 1:** Intraoperative and postoperative detection and diagnostic results of 13 patient lesion samples.

Sample	Intraoperative histopathological diagnosis	Intraoperative molecular diagnosis (AIGS)	Postoperative immunohistochemistry	Postoperative next‐generation sequencing	Postoperative correction diagnosis
1	Gliosis	IDH1 R132H mutation	IDH(−)	IDH1 R132H mutation	Lower‐grade glioma
2	Gliosis	IDH1 R132H mutation	IDH(−)	IDH1 R132H mutation	Lower‐grade glioma
3	Gliosis	IDH1 R132H mutation	IDH(−)	IDH1 R132H mutation	Lower‐grade glioma
4	Gliosis	IDH1 R132H mutation	IDH(−)	IDH1 R132H mutation	Lower‐grade glioma
5	Inflammatory changes	IDH1 R132H mutation	IDH(+/−)	IDH1 R132H mutation	Lower‐grade glioma
6	Gliosis	IDH1 R132H mutation	IDH(−)	IDH1 R132H mutation	Lower‐grade glioma
7	Gliosis	IDH1 R132H mutation	IDH(−)	IDH1 R132H mutation	Lower‐grade glioma
8	Gliosis	IDH1 R132H mutation	IDH(+/−)	IDH1 R132H mutation	Lower‐grade glioma
9	Inflammatory changes	IDH wild‐type	IDH(−)	IDH wild‐type	Inflammatory changes
10	Gliosis	IDH1 R132H mutation	IDH(−)	IDH1 R132H mutation	Lower‐grade glioma
11	Gliosis	IDH1 R132H mutation	IDH(+/−)	IDH1 R132H mutation	Lower‐grade glioma
12	Gliosis	IDH1 R132H mutation	IDH(+/−)	IDH1 R132H mutation	Lower‐grade glioma
13	Gliosis	IDH wild‐type	IDH(−)	IDH wild‐type	Gliosis

**FIGURE 4:**
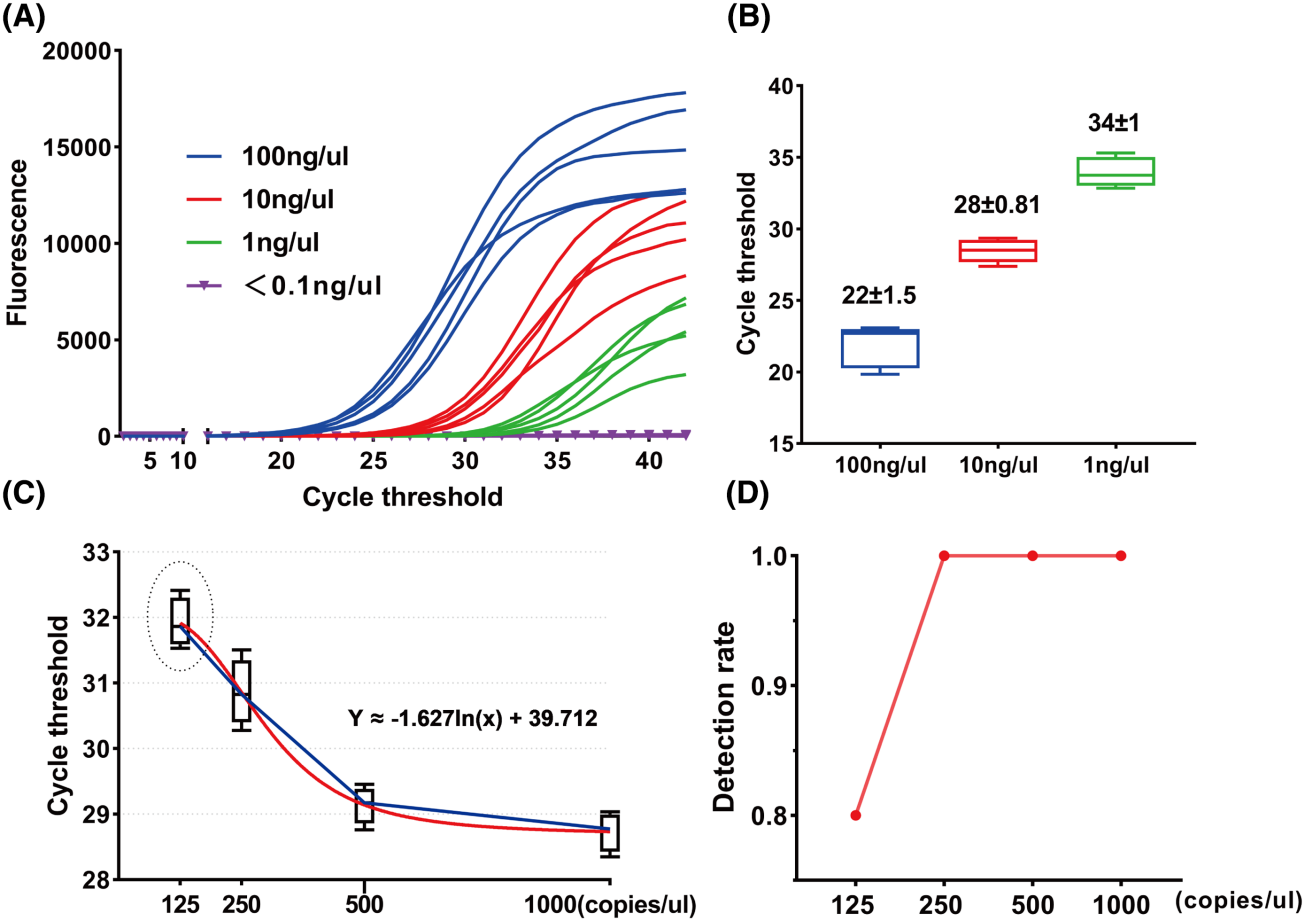
Estimation of the relationship between mutation abundance and threshold in UES‐LGG, as well as the prerequisites for accurate diagnosis. Real‐time fluorescence PCR detection was performed on five standard samples, and the fluorescence values of each gradient were plotted against the cyclic threshold (A). Comparison of average circulating threshold (CT) at different DNA concentrations (B). Diagram of the relationship between cycle threshold and gene copy number (C). The positive detection rate (D) corresponding to different gene copy numbers.

In order to explore the lower limit of mutation gene detection, we conducted DNA concentration detection on 5 IDH+ glioma samples and performed gradient dilution with dilution ratios of 1:1, 1:10, 1:100, and 1:1000, respectively. The final sample detection concentrations were controlled to be 100 ng/μL, 10 ng/μL, 1 ng/μL, and <0.1 ng/μL (Figure 4A). It was found that the higher the concentration, the shorter the time the fluorescence value reached the plateau period, and the smaller the CT threshold, which was within the effective threshold range (<40) A DNA content of < 0.1 ng/μL cannot accurately detect IDH mutations, which is related to the low abundance of IDH mutations (Figure 4B). Due to the need to detect mutations as accurately as possible in practical clinical applications to prevent misdiagnosis, we divided them into four groups again between two volumes of <0.1 ng/μL and 10 ng/μL: 125 copies/μL, 250 copies/μL, 500 copies/μL, and 1000 copies/μL. Research has found that as the copy number of genes increases, the cycle threshold decreases and can be fitted into a function curve, (cycle threshold) ≈ −1.627ln (copy number) +39.712 (Figure 4C), therefore, within this interval, we can roughly determine the gene copy number situation based on the cycle threshold. However, during the five AIGS tests conducted on each gradient, it was found that positive mutation results could not be detected every time the concentration was 125 copies/μL. Statistics showed that the detection rate at 125 copies/μL was 80% (Figure 4D). Therefore, we believe that when performing AIGS detection for IDH mutations during surgery, The minimum detection concentration to ensure a 100% detection rate is 250 copies/μL, which means that the minimum detection limit for accurately detecting “UES‐LGG” during surgery is 250 copies/μL.

## 5 DISCUSSION

Glioma, as the leading cause of death in young adults, urgently needs to be accurately identified and treated with the earliest and most effective methods.^5^ However, the identification of gliomas is not difficult in practice. In clinical practice, there are some LGGs with high differentiation and low heterogeneity that are difficult to distinguish from “gliosis” or “inflammatory changes”.^6^ These gliomas are often misdiagnosed and not treated effectively. Previous studies have shown that the misdiagnosis rate of intraoperative frozen sections in distinguishing gliosis from LGGs is 7%.^7^ Although many pathologists and neurosurgeons have acknowledged the difficulty in distinguishing between the two for many years,^1^ there has been no better way to timely and effectively distinguish between them. Most cases often obtain accurate pathological results after surgery, but in reality, neurosurgeons need to determine the type of lesion during surgery in order to make the most effective treatment. With the release of the 2021 CNS5 tumor classification standard, molecular diagnosis has brought benefits to the precise diagnosis and treatment of many tumors. At this time, many molecular markers of glioma have become the key to distinguishing tumors from non tumor diseases. IDH mutations do not exist in normal brain tissue. If IDH mutations are detected in samples of diseased tissue, gliomas are highly suspected.^6^, ^8^, ^9^ Similar mutations include TERTp, EGFR, and BRAF.

And how to quickly achieve molecular diagnosis during surgery seems to be a bigger issue. In recent years, with the continuous development of genetic testing technology, testing equipment has become more lightweight. Our research group has developed a fast molecular detection device that can be applied in surgery. The detection method based on real‐time fluorescence quantitative PCR has given it extremely high detection sensitivity and accuracy and has passed preliminary clinical validation.^4^ In this study, we applied it to the diagnosis of “UES‐LGG” and successfully identified 11 gliomas from suspected cases based on the presence of IDH mutations. All cases were validated by postoperative sequencing, reducing the misdiagnosis rate between gliosis and LGGs to 0%, greatly improving the surgical strategy of neurosurgeons. However, any detection method will have a limited detection range, and when it exceeds the range, accurate detection rates cannot be guaranteed. In this study, in order to clarify the detection threshold range of UES‐LGG, we performed gradient processing on the IDH+ sample of the standard sample. Finally, we believe that the detection accuracy based on AIGS is 80% when the detection sample concentration is 125 copies/μL, and 100% when the detection concentration is 250 copies/μL. Therefore, for UES‐LGG, the accuracy of detection can only be guaranteed when the DNA copy number detected on the sample is at least 250, But this does not mean that it cannot be detected below 250.

Therefore, we believe that timely detection of this type of tumor is necessary during surgery, which is of great significance for the formulation of surgical decisions and the survival prognosis of patients. In actual glioma surgery, we need to collect multiple samples from the tumor site under the meatus, conduct rapid histopathological evaluation, and perform intraoperative molecular diagnosis. When pathologists discover lesions with clear glioma properties, combined with molecular diagnosis results, we can obtain clear intraoperative diagnosis results; When pathologists are unable to detect lesions with clear glioma properties or consider the case to be in a borderline area (inflammatory changes or glial cell proliferation), intraoperative molecular diagnostic results are needed for auxiliary diagnosis. As long as molecular diagnosis reveals the presence of glioma‐related molecular expressions (IDH, TERTp), it can be considered as “UES‐LGG” and mutation abundance can be determined based on its circulation threshold. Subsequently, surgical resection can be performed according to glioma surgical standards.

### 5.1 Limitations

The main purpose of this study is to define the types of tumors that are difficult to distinguish between “gliosis” or “inflammatory changes” and LGGs. This type of tumor does not have typical glioma characteristics morphologically and is difficult to distinguish under the microscope, but it has clear glioma‐related molecular mutations. However, there is no clear concentration boundary between UES‐LGG and LGG. Therefore, we currently believe that when pathologists cannot distinguish between them through intraoperative frozen sections and postoperative immunohistochemistry, but positive mutations can be detected through intraoperative molecular diagnosis or postoperative sequencing, these “suspicious” cases are defined as “UES‐LGG”. Second, the overall number of cases involved in this study is relatively small, and we need more extensive case studies and clinical practice to validate the results in the future. We look forward to continuous technological innovation in the future, which can provide a more precise definition and identification of tumors in the “diagnostic boundary zone”, thereby reducing misdiagnosis. Surgical operators can make early response plans to reduce patient suffering.

## 6 CONCLUSIONS

In this study, we focus on a type of “problem” that is frequently encountered in clinical work. Although the problem is small, it is like a thorn in the throat. We defined this type of tumor that is prone to misdiagnosis using UES‐LGG. Due to its extremely low positivity and mutation abundance, it is difficult to detect by immunohistochemistry or even next‐generation sequencing. Moreover, this type of tumor requires rapid identification of its properties during surgery, which traditional detection methods do not have. With the emergence of qRT‐PCR‐based intraoperative rapid molecular diagnostic technology, it will greatly solve the current difficulties, compensate for the errors caused by manual detection, improve the detection rate of intraoperative diseases, and provide a theoretical basis for more accurate surgical plans for surgeons.

## AUTHOR CONTRIBUTIONS

Experimental design: Zhe Han, Hao Xue, and Gang Li. Experimental methods: Qingtong Wang, Jia Li, Huizhong Chi, and Caizhi Ma. Data analysis: Zhe Han, Deze Jia, Mei Qi, Xueen Li, Kailiang Zhang and Zichao Feng. Manuscript‐writing: Zhe Han, Qingtong Wang, and Hao Xue. Zhe Han and Qingtong Wang authors contributed equally to the manuscript.

## FUNDING INFORMATION

This work was financially supported by Natural Science Foundation of Shandong Province of China (award number ZR2021LSW025), the Fundamental Research Funds for the Central Universities (award number 2022JC019), Jinan Science and Technology Bureau of Shandong Province (award number 2021GXRC029), Shandong Province Youth Innovation Plan (award number 2022KJ011), Natural Science Foundation of Shandong Province of China (award number ZR2023LZL004), National Natural Science Foundation of China (award number 82072776, 82273195), National Natural Science Foundation of China (award number 82072775, 82273286), Science and Technology Innovation 2030‐“Brain Science and Brain‐Like Intelligence Technology” (award number 2021ZD0201600), Taishan Pandeng Scholar Program of Shandong Province (award number tspd20210322) and Youth Taishan Scholar Program of Shandong Province (award number tsqn202211316).

## CONFLICT OF INTEREST STATEMENT

The authors declare that they have no conflict of interest to disclose.

## Supporting information


Table S1

## Data Availability

The data that support the findings of this study are available from the corresponding author upon reasonable request.
