# Supplementary material for: Ultra‐early stage lower‐grade gliomas: How can we define and differentiate these easily misdiagnosed gliomas through intraoperative molecular diagnosis
Source: CNS Neurosci Ther. 2024 Oct 9;30(10):e70044. doi: 10.1111/cns.70044 (PMC11464210; doi:10.1111/cns.70044)
Supplement: Supplementary file 1 — Table S1 [file CNS-30-e70044-s001.docx]

| Supplementary Table 1：Clinical basic information and follow-up information of 13 patients | | | | | | | |
| --- | --- | --- | --- | --- | --- | --- | --- |
| **NO.** | **Gender** | **Age（y）** | **Intraoperative histopathological diagnosis** | **Final diagnosis** | **Follow-up time（m）** | **Recrudescence** | **Survival status** |
| 1 | M | 35 | Gliosis | Astrocytoma | 47.00 | No | Live |
| 2 | F | 44 | Gliosis | Astrocytoma | 44.63 | No | Live |
| 3 | F | 48 | Gliosis | Oligodendroglioma | 44.47 | No | Live |
| 4 | F | 49 | Gliosis | Astrocytoma | 44.00 | No | Live |
| 5 | M | 45 | Inflammatory changes | Astrocytoma | 42.33 | No | Live |
| 6 | M | 55 | Gliosis | Astrocytoma | 39.77 | No | Live |
| 7 | F | 46 | Gliosis | Astrocytoma | 39.77 | No | Live |
| 8 | F | 42 | Gliosis | Astrocytoma | 39.63 | No | Live |
| 9 | F | 27 | Inflammatory changes | Inflammatory changes | 38.80 | No | Live |
| 10 | M | 44 | Gliosis | Oligodendroglioma | 38.07 | No | Live |
| 11 | F | 45 | Gliosis | Astrocytoma | 38.03 | Yes | Live |
| 12 | M | 36 | Gliosis | Astrocytoma | 37.43 | No | Live |
| 13 | M | 43 | Gliosis | Gliosis | 37.30 | No | Live |
